# Organizational conditions for engagement in quality and safety improvement: a longitudinal qualitative study of community pharmacies

**DOI:** 10.1186/s12913-018-3607-7

**Published:** 2018-10-17

**Authors:** Denham L. Phipps, Christian E. L. Jones, Dianne Parker, Darren M. Ashcroft

**Affiliations:** 1 Division of Pharmacy and Optometry, The University of Manchester, Manchester Academic Health Science Centre, Oxford Road, Manchester, UK; 2 NIHR Greater Manchester Patient Safety Translational Research Centre, The University of Manchester, Manchester Academic Health Sciences Centre, Oxford Road, Manchester, UK

**Keywords:** Quality and safety, Improvement, Behavioural change, Pharmacy, Primary care, Healthcare organizations

## Abstract

**Background:**

While efforts have been made to bring about quality and safety improvement in healthcare, it remains by no means certain that an improvement project will succeed. This suggests a need to better understand the process and conditions of improvement. The current study addresses this question by examining English community pharmacies attempting to undertake improvement activities.

**Method:**

The study used a longitudinal qualitative design, involving a sample of ten community pharmacies. Each pharmacy took part in a series of improvement workshops, involving use of the Manchester Patient Safety Framework (MaPSaF), over a twelve-month period. Qualitative data were collected from the workshops, from follow-up focus groups and from field notes. Template analysis was used to identify themes in the data.

**Results:**

The progress made by pharmacies in improving their practice can be described in terms of a behavioural change framework, consisting of contemplation (resolving to make changes if they are required), planning (deciding how to carry out change) and execution (carrying out and reflecting on change). Organizational conditions supporting change were identified; these included the prioritisation of improvement, a commitment to change, a trusting and collaborative relationship between staff and managers, and knowledge about quality and safety issues to work on.

**Conclusions:**

Our study suggests a process by which healthcare work units might undergo improvement. In addition to recognising and providing support for this process, it is important to establish an environment that fosters improvement, and for work units to ensure that they are prepared for undergoing improvement activities.

## Background

Quality and safety improvement is an endeavour that often begins with good intentions but does not always end as expected or desired. Many of the projects documented in the healthcare literature report mainly positive results, with regard either to climate or to performance. [1–5] However, a healthcare organization that seeks to embark on an improvement journey may well encounter difficulties in negotiating the territory. As a case in point, Dixon-Woods et al. [6] observed that staff across the English National Health Service felt disempowered by issues such as uncertainty, poor systems, high workloads, and a shortage of resources; all of which may serve to affect their engagement in improvement activities. Similar observations have been made in other studies [7, 8]; to these issues they add the effect of cultural factors such as staff participation, attitude towards organizational learning, and working relationships.

Hence, it is important to understand how contextual and process factors affect the implementation of quality and safety improvement. Given the importance of a top-down approach to improvement, this question might usefully be addressed from the perspective of strategic or senior managers within a healthcare organization [9, 10]. However, it would also be of interest to consider how improvement is experienced at the frontline of care delivery, in work units. Vogus and colleagues [11, 12] note that, while safety improvement may be enabled by managers and institutional policies, it is enacted by frontline staff and evolved as a result of this enactment. Similarly, Moore & Buchanan [13] suggest that the success of frontline work units in dealing with local, small-scale quality and safety issues may drive broader cultural change.

The aim of the study reported here was to examine the process and conditions associated with safety improvement in the community pharmacy setting. Internationally, community pharmacies are responsible for the effective and safe supply of medication in the primary care sector, which in turn accounts for a large proportion of medicines usage within the healthcare system. [14] Empirical data estimating that medicines usage accounts for 3–9% of hospital admissions across a number of countries [15], and incurs a harm rate of 3% in English primary care [16], point to the safety-critical nature of primary care medicines management [17]. Within this system, community pharmacies have been estimated to encounter a prevented dispensing error rate of approximately 0.2 to 0.4% in the United Kingdom, and their United States counterparts 1.2% [18]; in addition, community pharmacies are increasingly contributing to the provision of other primary care services, such as medication reviews and health promotion, whilst operating as a commercial enterprise, all of which adds to the complexity of their work [19–21]. A recent study of community pharmacies in Great Britain characterised them as typically incurring a high workload, with a tension between providing resources to manage this workload and maintaining commercial viability; safety culture in a given pharmacy being a product of the way in which it manages this relationship [22, 23]. So, it would be of value to understand what is needed for successful improvement efforts in community pharmacies – both to ensure that they make an effective contribution to primary care quality and safety in their own right, and to identify general insights about improvement that might be applicable to other areas.

## Method

### Participants

Our study used a longitudinal qualitative design. We invited ten community pharmacies, all within the sampling frame of a metropolitan county in North-West England, to participate in a quality and safety improvement intervention over a twelve-month period between June 2014 and May 2016. We sought to select pharmacies on a purposive basis to represent the typical range of sizes and organizational structures (that is, pharmacies with a small number of staff versus those with a large number of staff, and independently-run pharmacies versus pharmacies that were part of a network or “chain”). Because we planned to collect a substantial amount of qualitative data from each pharmacy, we limited the number of pharmacies recruited to that which was sufficient to provide a breadth of characteristics. Pharmacies came into the sample through several routes: some responded to an advertisement circulated by the county’s local pharmaceutical committees and local professional networks; others volunteered for the study through a national “research-ready pharmacies” scheme; while others (most of the chain branches) were approached by us having been nominated by their respective pharmacy head offices. In each pharmacy, the pharmacy manager and all members of staff gave individual informed consent to take part in the study following telephone contact and an initial face-to-face discussion with a member of the study team. Individual participants received gift vouchers and expenses in return for taking part in planned workshops, focus groups and individual interviews. Of the pharmacies directly approached to take part, one declined due to concerns about the time commitment required, and another took part but did not complete the study due to ceasing operation during the study period. The latter pharmacy has been retained in the study sample.

### Procedure

The intervention used the community pharmacy version of the Manchester Patient Safety Framework (MaPSaF) [24, 25]. This is based on the cultural development model proposed by Westrum [26] and elaborated by Parker et al. [27], which characterises an organization’s culture in terms of the way in which it elicits and handles information about quality and safety. It presents descriptors of a healthcare organization at successive levels of quality and safety development (“pathological”, “reactive”, “calculative”, “proactive” and “generative”). The descriptors are arranged into eight domains: overall commitment to quality and safety; incident causation and reporting; investigation of patient safety incidents; learning from patient safety incidents; communication about safety issues; staff management and safety issues; education and training about safety; team working around safety issues. An example of the descriptors for one of the domains, illustrating the successive developmental levels, can be seen in Table 1. Staff members can assess their own pharmacy site and (where relevant) pharmacy chain by comparing it against the descriptors for each level and domain. The MaPSaF is intended to stimulate individual and collective reflection on relevant beliefs and practices, and hence serves as a learning and development aid.

**Table 1:** MaPSaF descriptors for “learning following an incident”

Level	Excerpt from descriptor
1 (“Pathological”)	No attempts are made to learn from incidents unless imposed by the pharmacy inspector.
2 (“Reactive”)	Little if any learning occurs, and what does occur is related only to the level of inconvenience experienced by the manager or head office.
3 (“Calculative”)	Some systems are in place to facilitate learning, but lessons are confined to specific local changes and not communicated across the organization.
4 (“Proactive”)	The pharmacy has a learning tradition and systems exist to share learning, such as reflection and audit. Members of staff are actively involved.
5 (“Generative”)	The pharmacy is committed to sharing learning and information across the organization. Improvements occur without being triggered by an incident.

The study began with a MaPSaF workshop at each of the pharmacies. During the workshop the participants read the descriptors and, firstly individually and then in group discussion, appraised their pharmacy with respect to the depicted developmental levels (following initial pilot testing, the original labels for the levels had been replaced with numerical labels 1 to 5 in order to avoid biasing the participants’ responses). Once they had done so, the facilitators anonymised then summarised all participants’ responses before reporting them back to the group. In doing so, the facilitators highlighted domains in which the pharmacy was consistently classified at either the higher or lower developmental levels, and domains in which the classifications were spread across the different levels (the latter indicating a lack of agreement amongst participants). The participants were then invited to comment on the responses as the opening to a broader discussion about quality and safety in their pharmacy. Finally, the participants identified and agreed on actions that they might carry out to improve quality and safety. Subsequent MaPSaF workshops were held after six months and twelve months; on each occasion the participants discussed any changes that had occurred in the pharmacy since the previous workshop. In addition to the workshops:

- each pharmacy was visited by the researchers twice after the first workshop (one during the first month and once during the second month). The purpose of each visit was to obtain informal data about subsequent learning and implementation of improvement activities, as well as the context within which these were taking place;
- a focus group was held with the workshop participants at each pharmacy, three months after the first and second workshops. The purpose of the focus group was to discuss participants’ experiences of the preceding workshop and identify any immediate learning or actions that had resulted from it.

The workshops and focus groups were facilitated by DLP (an occupational psychologist) and CELJ (a research psychologist and pharmacy dispenser). With the permission of the participants, all of these were audio recorded and transcribed, and the facilitators took field notes to elaborate on the process of the workshops. DLP and CELJ also carried out the pharmacy visits, from which they took further field notes. During the data collection period, the facilitators regularly reviewed the accumulating data to ensure that they were being elicited and recorded in a consistent manner, guided by the study’s objective to understand the experience of planning and implementing improvement activities, and the organizational context in which it was occurring. At the end of the data collection period, the transcripts and field notes were combined to create the dataset for analysis.

### Data analysis and results

The data were analysed using template analysis [28]. Template analysis is an iterative process that involves the analyst reading through qualitative data and creating a “template” consisting of the themes that emerge from the reading. The template is then modified and extended through successive readings until it provides sufficient coverage of the data. In the current study, DLP and DP (the latter a social psychologist who had been involved in the development of MaPSaF, but not the data collection for the current study) carried out the analysis, starting with a general theme of “making changes to improve patient safety”. During the analysis, they compared participants’ accounts between the participating pharmacies, and between time points within each pharmacy, in order to generate subordinate themes. Each theme was then developed further by successive reading of the data, until no new themes were generated. Following initial reading of the data it became apparent that the concept of individual and group behaviour change, as distinct from the organizational-level development model underlying MaPSaF, would be relevant to the analysis; this led the analysts to consult health behaviour literature to assist with interpretation of the emerging themes. Version 10 of the NVivo computer program was used to document the analysis. The final template was reviewed by CELJ and DMA (the latter a pharmacist who had been involved in the adaptation of MaPSaF for community pharmacies, but not the data collection for the current study) to ensure that it accounted for the data and adequately addressed the research question. In order to obtain member validation of the findings we invited seven community pharmacists who did not participate in the study, but who were drawn from the same sampling frame, to review and comment on the template. This group made additional observations about improvement activities in this setting, which informed our interpretation of the findings.

## Results

In total, 50 members of pharmacy staff (14 pharmacists and 36 members of support staff) took part in the study between June 2014 and May 2016. The characteristics of each pharmacy, and the distribution of staff between the pharmacies, are shown in Table 2.

**Table 2:** Characteristics of the pharmacies included in the study

Pharmacy	1^a^	2	3	4	5	6	7	8	9	10
Organization^b^	Chain A	Independent	Chain B	Independent	Chain C	Chain D	Chain C	Chain E	Chain D	Chain C
Staffing:										
- Pharmacist	1	1	1	4	1	2	1	1	1	1
- Support staff	6	1	3	9	2	2	4	4	3	2

^a^Pharmacy 1 withdrew from the study following the first session, due to ceasing operation

^b^Chains A and B operate in the study region only; Chains C, D and E operate nationwide

### General observations

Over the course of the study we noted some variation between the pharmacies, in terms of both their engagement with the intervention and their identification and implementation of change actions. Examples of the changes identified during the workshops included:

- Introducing communication aids to facilitate team coordination (for example, whiteboards and notebooks);
- Using team meetings, briefings or one-to-one sessions to discuss patient safety issues;
- Making better use of incident reporting systems, for example by sharing responsibility for reporting across the whole team;
- Reorganising or tidying the workspace;
- Establishing a formal training programme for new staff;
- Reallocating tasks across staff members, times or locations.

As shown in Table 3, we identified two main themes from the data. These themes were labelled “doing quality and safety improvement” and “facilitating quality and safety improvement”, and each included sub-themes.

**Table 3:** Themes and subthemes identified in the data

Themes	Description
Doing improvement	The process of quality and safety
Contemplating change	Thinking about / discussing whether change is needed
Preparing for change	Thinking about / discussing what would be needed to bring about change
Behavioural intention	The formation of behavioural intentions
Implementation intention	The formation of implementation intentions
Implementing change	Executing behavioural and/or implementation intentions, and reviewing their effects
Facilitating improvement	What helps or hinders quality and safety improvement?
Prioritisation	Making quality sand safety improvement a priority relative to other objectives (e.g. productivity)
Involvement	How much involvement staff members and managers have in improvement
Relationships	How well the staff and managers work together (e.g. hierarchy gradient; communication)
Knowledge	Having access to knowledge about quality and safety issues, and how to address them

### Doing quality and safety improvement

The progress made by each pharmacy in reviewing and modifying its practice appeared to resemble a process similar to the “stages of behavioural change” that have been suggested for individual and organizational behaviour. [29, 30] We identified four subthemes in our data, each relating to the suggested process. The first, *contemplating change*, refers to participants discussing whether change was needed in the first place.

*“I think we fail on writing incidents up. […] I think we’re brilliant with [near misses], but […] incidents that leave the shop, I do think we’re quite poor at.”* [Pharmacy 4, Workshop 2]

The second subtheme, *preparing for change*, refers to participants discussing what would be needed to bring about change. Drawing from the literature on health behaviour, we identified two further subthemes. One refers to participants forming an intention to carry out a specific behaviour (*behavioural intention*), and the other refers to participants specifying how they will see through their intention in the face of any anticipated barriers (*implementation intention*). [31, 32]

*“What we’re probably going to do is […] have a bulletin or something like that where we can put a bit more detail in and […] a section for learning.”* [Pharmacy 2, Workshop 2]

*“The only thing people can do [if they are short of time] is maybe make a quick note of it in the diary, like a reminder […] saying near miss log or what it was […], just to remind them, and they could fill [the incident report] in later.”* [Pharmacy 8, Workshop 2]

The third subtheme, *implementing change*, refers to pharmacy staff executing changes to their practice or to their work environment, and reviewing the effects of these changes.

*“I think we learned a lot from the controlled drug errors. […] We changed quite a lot in how we dispense [controlled drugs] and we’ve [now] got special baskets for them […], so I think we learnt quite a lot from that.”* [Pharmacy 4, Workshop 2]

During their participation in our study, the pharmacies varied in the extent to which they demonstrated each of these subthemes. At some pharmacies, most of the subthemes appeared to be present in participants’ discussions and actions. Correspondingly, these pharmacies made more obvious progress in enacting quality and safety improvement, identifying issues to work on and making efforts to address these issues. For example, in Pharmacy 6, the discussion in the initial workshop led the participants to reflect on the quality of task-related communication (for example, relating to handoffs and stock status), and conclude that it needed to be improved. They considered alternative communication tools, before deciding to set up a whiteboard in the dispensary. In subsequent workshops, the participants reflected on the positive effect of the whiteboard in assisting communication between them.

While our presentation of the subthemes implies a linear process from contemplation to implementation, however, we found progression between these subthemes to be more iterative in practice. For example, prior to taking part in the study the manager of Pharmacy 4 had already contemplated the need to deal with distractions in the dispensary, and installed an automated telephone answering system to field incoming calls that were one source of distraction. However, he had then been unable to configure the system in a suitable manner. He did not, though, have a clear view about how to replace this system at the time of the first workshop, and so part of the discussion was to identify alternative, non-technological solutions that could be implemented in the meantime, such as a staff rota for answering the existing telephone. The latter line of conversation resulted in the suggestion to divide work between the existing dispensary and a second, previously underused, one that had fewer distractions; this suggestion was subsequently implemented during the course of the study, while the plan for a new telephone system was completed by the time of the final workshop. It appeared though that, in the absence of the new system, the pharmacy’s attempts to reorganise work were having a beneficial effect in themselves.

*While the error reports haven’t been analysed in detail, one pattern that the pharmacist has noticed is that fewer errors are occurring during an early morning session (starting at 7 am) that was recently started in order to deal with the workload. Could this be due to there being fewer distractions (*e.g. *phones ringing) during this session?* [Pharmacy 4, Field note from site visit]

Meanwhile, about half of the pharmacies in our sample demonstrated few or none of the activities described in these subthemes; some contemplated the need for change but either did not form any specific intentions or did not subsequently enact their intentions. There was, as a consequence, relatively little progress in identifying and making quality and safety improvements. For example, in the first workshops at Pharmacies 3 and 5, the participants recognised the importance of improvement activities but felt unable to identify any actions on their part, as they attributed their safety issues primarily to a lack of resources that was seen as the responsibility of the head office to resolve. Meanwhile, in Pharmacy 7, there was little evidence of contemplating, identifying or enacting improvement actions. The following observation was typical of the workshops at this pharmacy.

*The support staff went into a side room with us for the workshop, but the pharmacist remained outside, working at the dispensary counter. During the workshop, the pharmacist called to the support staff to hurry up as they needed to get back to work.* [Pharmacy 7, Field note from first workshop]

We also noted that the extent to which contemplation, planning and implementation occurred varied within the pharmacies as well as between them. For example, as mentioned earlier, the staff at Pharmacy 6 took steps to resolve their problems in communication. Their initial workshop, though, also revealed inconsistencies between the participants regarding their use of the company’s near-miss reporting system. While they contemplated the need for a consistent approach to incident reporting, their planning and implementation of such an approach was less obvious.

*The manager told us that staff were getting better at near-miss reporting following the first workshop. However, I didn’t really get a clear answer as to whether there was now agreement between all members of staff about using the near-miss form. The form is on the counter, so all staff can readily access it.* [Pharmacy 6, Field note from post-workshop visit]

### Facilitating quality and safety improvement

The data suggested a set of characteristics that either aided or hindered the pharmacies in contemplating, planning and implementing quality and safety improvement. Some of these characteristics reflected existing practice within the pharmacy. For example, participant accounts alluded to the perceived priority of quality and safety activities in the face of productivity demands.

*“You might just be busy at the time. So if we’ve got a shop full of people you’ve not really got time to [stop], and [reporting a near-miss] might just get forgotten about because you might be occupied with something else.”* [Pharmacy 8, Workshop 2]

As with almost all of the participants in the study, the participants in Pharmacy 8 expressed a belief that, in principle, safety was a high priority. However, as this excerpt indicates, they (again like the other pharmacies) found their task demands a barrier to engagement in safety improvement activities. Indeed, most of the pharmacies were reluctant to cease work activities completely during the workshops, preferring instead to divide their attention between the exercise and their ongoing work. Some of the pharmacies, however, did manage to allocate time away from their work (either by organising staff cover or by staying behind at the end of the working day) to concentrate on the exercise, which allowed them to engage with it more fully. Even in these pharmacies, though, participants were conscious of production pressure in their work that might impede their engagement in improvement activities.

*“People having their own training programme [for example], that’s [a lot of] time input, and [in] an ideal world that’s great, but pharmacy is really skinny with staff, we really work every minute. Like now, we’re itching to do [medicine] trays right now.”* [Pharmacy 5, Workshop 2]

The pharmacies also varied in their inclusion of staff members and managers in quality and safety activities. In some of the pharmacies, the participants were explicit about the need for all staff members to play a role.

*“[We have to] be clear in our working ethic, clear in our procedures but also actually think about general safety as part of your day to day practice. [For example, you see a box left out]. We’ll question it; why’s this box left out, why’s this cream stood there, why is it not in the cream drawer, why is this here, that kind of thing”* [Pharmacy 2, Focus group 2]

The proactivity to which the pharmacist at Pharmacy 2 aspires could be contrasted with the experience of participants at Pharmacy 7, whose efforts at risk reduction were triggered by a visit from the pharmacy chain area manager. While the participants here had managed to sustain the change in practice, it was unclear whether there had been any broader effect of the manager’s intervention.

*“Our area manager came in and she [said] we had to keep up to a certain […] standard. […] We had […] boxes lying about, papers and stuff like that. We had to clear that up and [maintain] a clean desk where we can actually dispense, so avoiding the risk of errors occurring, so that’s what we did.”* [Pharmacy 7, Workshop 1]

The participants’ accounts further highlighted the importance of leader involvement – either to inspire or to reinforce staff members’ efforts – in stimulating quality and safety improvement. For example, the role of the pharmacy manager is referred to by participants from Pharmacies 3 and 4.

*“I could tell the [previous] manager things, if I thought something wasn’t working I […] say, right, this isn’t working, we need to do this and this. But […] because [our new manager] is new to the company he’s learning as well as he goes along. So although we’re learning off him, he’s learning off us as well.”* [Pharmacy 4, Workshop 2]

*“[When the current pharmacist] came here, what I thought was good [was when she] changed the baskets [so that they were now organised by] colour, [because] we didn’t have that before.”* [Pharmacy 3, Workshop 1]

The experience of Pharmacy 3 is particularly noteworthy. This pharmacy had several changes of pharmacy manager and staff during the course of the study, which affected the pharmacy’s engagement with the exercise; in each workshop, the pharmacy team was in effect working from scratch. As both quotes show, staff turnover can have a positive effect on quality and safety (due to the introduction of new ideas and fresh impetus) but it can also disrupt collective learning or limit the staff’s commitment to long-term improvement.

A further aspect of staff and leader involvement in improvement related to the quality of the interaction between them. Many participants referred to the need for open two-way communication about quality and safety issues, unaffected by a hierarchy gradient or feelings of threat or shame. To take Pharmacy 4 as an example, some of the participants remained relatively quiet in the initial workshop, but made more contribution to later workshops. During the later discussions, it emerged that a barrier to them speaking in earlier sessions (aside from their unfamiliarity with the exercise) was the presence of other members of staff who they believed would respond negatively or even punitively to any input they made.

*“It does make it awkward when […] they know that the situation you’re talking about involves them. […] You can […] feel them looking at you and you [think] ‘should I have said this?’”* [Pharmacy 4, Workshop 2]

During their workshops, the staff at Pharmacy 6 discussed their inconsistent use of the company’s formal near-miss reporting system, which continued throughout the study. They explained that they preferred to discuss any near-misses amongst themselves, due to a concern that the formal system had little value for learning but did impose unnecessary bureaucracy and expose staff to the risk of being sanctioned.

*“[I prefer] word of mouth […] because I’m not into getting people in trouble. I just want to say, […], just discreetly […] this is what’s happened. You know what, it’s actually a bit embarrassing […]. Don’t you think so?”* [Pharmacy 6, Final workshop]

A final sub-theme that related to the facilitation of improvement was the pharmacy having sufficient knowledge about what issues might need to be addressed and how. While the workshops allowed the exchange of ideas between members of staff in this regard, the accounts from some participants indicated the value of a permanent knowledge base, whether compiled from internal or external sources. For example, the manager of Pharmacy 1 felt that the lack of information sharing between pharmacies in her chain was a barrier to improvement.

*“We get no communication [about] what’s happening in [the] other branches […]. We have 20 shops in the company now, but I don’t hear about [the] common errors […] or any incidents, which I think we could learn from.”* [Pharmacy 1, Workshop 1]

Despite this, the manager took the opportunity to share knowledge of her own; she allowed us to pass details of her in-house staff training onto Pharmacy 2, which had identified staff training as an improvement action. The manager of Pharmacy 2 further reflected on the need to constantly seek information about quality and safety issues.

*“There’s always, you know, you can never know enough. We can’t ever know enough, there’s always something that you can learn from.”* [Pharmacy 2, Workshop 2]

## Discussion

Our study has examined the process by which quality and safety improvement occurs within community pharmacies, and the conditions under which it occurs. We found that the process could be characterised in terms of contemplating, planning and executing change, analogous to a “stages of behavioural change” framework. Within this process, explicit consideration of behavioural and implementation intentions appeared to be helpful in planning for improvement activities, as did having sufficient knowledge about which issues to address and how. With regard to the conditions for change, participants referred to the prioritisation of quality and safety improvement relative to other organizational objectives, most notably maintaining productivity. Those pharmacies that made the most progress tended to demonstrate a commitment to change on the part of managers and staff, and a working relationship between staff and managers that fostered greater trust and collaboration.

Previous studies of quality and safety improvement, both in healthcare and elsewhere [33], have highlighted the need to understand the mechanisms and context of improvement, as opposed to looking only at the technical nature of an improvement intervention. Our findings suggest some of the mechanisms and contextual factors that are relevant to quality and safety improvement at a local level. These factors may explain the variation that has sometimes been observed in the engagement of work units with an improvement intervention, for example by Vigorito et al. [34]

Our findings suggest some further issues regarding safety improvement. Firstly, they appear to echo the notion of organizational readiness for change as described by Weiner [35], in that they suggest the need for a pharmacy to possess both commitment and efficacy for change to occur. In other words, it is important to consider how well prepared a given pharmacy is to undergo change.

A second issue, also echoing previous studies [6, 22, 36, 37, 38], is that organisational structures, resources and policies can either stimulate or constrain quality and safety improvement at the frontline. Implicit in the contextual factors described here is a relationship between decisions and practices at the “blunt end” (head offices or pharmacy owners) and those at the “sharp end” (pharmacy staff) regarding the provision and use of resources. For example, the ability of a pharmacy to focus on safety issues can depend on the resources that the head office decides to allocate to it, as well as the organizational systems that shape how these resources are used. Alternatively, a given pharmacy’s approach to safety issues can be modelled or directly challenged – for better or for worse – by local and senior managers. As Valentine [38] demonstrates, the deliberate alignment of local managers’ activities with organizational goals would (assuming the latter are clear and appropriate) facilitate top-down improvement. Furthermore, that frontline worker participation and psychological safety should be proposed as conditions for change supports the arguments made by Macrae [39] and Leonard & Frankel [40] respectively. They also point to the role of organizational culture in determining engagement with improvement activities, for example as observed in relation to incident reporting [41].

The final issue highlighted by our findings is the complexity of quality and safety improvement. As the pharmacies in our study exemplify, and as noted previously [42], the stages of change are not necessarily visited in the linear fashion that is implied by their presentation. The participants’ experiences also illustrate the slow pace of change and the need to attend to structural issues; even more so if the aim is to go beyond the immediate local practices and bring about change more widely [43, 44]. Regarding structure in particular, a recently proposed model of emergent organizational change [45] notes the importance of managerial sense-making in identifying and amplifying local improvements across the organization.

Our study data have some limitations that should be taken into consideration. The participating community pharmacies were sampled from one geographical area of England. Also, the independently-run pharmacies had self-selected into the study, and therefore might have been more motivated to make use of our intervention than other similar pharmacies. However, all of the pharmacies were selected on a purposive basis to represent a range of community pharmacy organizational structures. As a result, our findings are likely to apply to other pharmacies that were not part of the sampling frame. A key strength of our study design is that it allowed us to examine how attempts at improvement proceed or stall over a period of time, and to identify how naturally-occurring circumstances affect improvement activities in pharmacies. These insights may help to account for observed change or lack of change in quality and safety measures during a given measurement period.

There are several important implications of our study. From a theoretical point of view, our study suggests a link between quality and safety improvement and models of behavioural change, such that the latter may provide a useful framework for stimulating or supporting improvement activities. Regarding methodology, our study demonstrates the value of longitudinal qualitative data (alongside quantitative or cross-sectional data) to gain further insights into the context and mechanisms of improvement activities. Our findings indicate aspects of organizational design and functioning that would set the scene for improvement. Staff at the frontline could be directly supported through the process of change – for example, through the provision of learning resources or through a targeted coaching intervention [46]. However, they also need to be in conditions that provide the capacity for them to participate in quality and safety improvement. Hence, as a precursor to improvement work, those organizations responsible for the running of pharmacies should ensure that the pharmacies have sufficient resources, time and incentive to engage in improvement activities. Meanwhile, the pharmacies themselves should reflect on their ability to engage in improvement, if necessary developing their team stability, openness, knowledge and commitment to improvement. Finally, we would suggest that future research, whether in pharmacies or in other healthcare settings, further explores the context and process of change in order to better understand the effect of quality and safety improvement interventions; on the basis of our findings, factors that are likely to be relevant for a given organization include the operational circumstances, the support provided by managers at all levels, the motivation of staff to undertake improvement activities and the ability to set and implement goals for improvement.

## Conclusion

In order to ensure the success of quality and safety improvement activities, it is important to understand how healthcare work units engage in improvement, and what can facilitate or hinder their engagement. Using qualitative data from community pharmacies, we have explained their engagement in terms of a behavioural change process, and identified a set of organizational conditions that would allow them to successfully carry out improvement activities. These suggest ways in which healthcare organizations can help work units to prepare themselves for quality and safety improvement.

## Abbreviation

MaPSaF: Manchester Patient Safety Framework
